# Depression in healthcare workers from the COVID-19 Care and Isolation Center - Villa Panamericana: a single-center prospective study in Peru

**DOI:** 10.31744/einstein_journal/2022AO6707

**Published:** 2022-04-13

**Authors:** Jeel Moya-Salazar, Walter Saciga-Saavedra, Betsy Cañari, Hans Contreras-Pulache

**Affiliations:** 1 Hospital Nacional Docente Madre Niño San Bartolomé Lima Peru Hospital Nacional Docente Madre Niño San Bartolomé, Lima, Peru.; 2 Escuela de Medicina Universidad Norbert Wiener Lima Peru Escuela de Medicina, Universidad Norbert Wiener, Lima, Peru.

**Keywords:** COVID-19, Coronavirus infections, SARS-CoV-2, Betacoronavirus, Depression, Health personnel, Mental health, Peru

## Abstract

**Objective:**

Depression is a mental problem that affects the well-being of healthcare workers, impacting the quality of care and even leading to commit suicide. We aim to the levels of depression in frontline healthcare workers during the first severe acute respiratory syndrome coronavirus 2 (SARS-CoV-2) outbreak in Peru.

**Methods:**

A prospective cohort study was designed in the coronavirus disease 2019 (COVID-19) Care and Isolation Center – Villa Panamericana in eastern Lima. Care and Isolation Center-Villa Panamericana houses about 150 healthcare workers and COVID-19 patients. The Montgomery-Asberg Depression Rating scale was used for depression assessment.

**Results:**

A total of 96 participants (30±5.6 years) were analyzed: 15 (15.6%) physicians, 39 (40.6%) nurses, 14 (14.6%) medical technologists, and 28 (29.2%) nurse technicians. Mild, moderate, and severe depression were present in 35 (36.5%), 44 (45.8%), and 9 (9.4%) of the cases, respectively. The physicians and nurses reported more severe levels of depression: 8 (53.3%) physicians and 18 (46.2%) nurses presented moderate depression; and 2 (13.3%) physicians and 3 (7.7%) nurses presented severe depression (p=0.005). This study determined greater symptoms of depression according to years of work (p=0.001). Thirty-two healthcare workers had COVID-19, 4 (12.5%) physicians, 9 (28.1%) nurses, 7 (21.9%) medical technologists, and 12 (37.5%) nurse technicians. Twenty-four (75%) participants showed symptoms of COVID-19 and developed moderate (12 [37.5%]) and severe (3 [9.4%]) symptoms of depression (p=0.041).

**Conclusion:**

This study clearly demonstrated a high prevalence of depression in the Care and Isolation Center-Villa Panamericana frontline healthcare workers during the COVID-19 pandemic in Peru.

## INTRODUCTION

Due to the sudden large-scale expansion of severe acute respiratory syndrome coronavirus 2 (SARS-CoV-2), several countries have chosen different degrees of confinement and social isolation to reduce the infection rate and mortality of this new plague.^(1,2)^ The impact of sudden changes in daily activities and the uncertainty of the spread of the virus have become the pillars of the global mental health crisis.^(3)^ This has brought an increase in mental disorders with a high prevalence of suicidal tendencies, depression, anxiety, and stress, especially in the middle and post-pandemic phase.^(4)^

Fear of infection, infodemic, boredom, and economic issues are factors causing negative psychological effects in the general population. Healthcare workers may also be severely affected by various stressful situations related to their role in the care of coronavirus disease 2019 (COVID-19) patients.^(5,6)^ This sector is more vulnerable, since direct contact, as well as the care of suspected and confirmed cases of COVID-19, makes them more likely to develop greater mental problems. In addition, they have to work uninterrupted and long hours, due to the increasing demand of patients and the lack of healthcare workers staff and personal protective equipment.^(7)^

Depression is a mental problem that affects the well-being of healthcare workers, impacting the quality of care and even leading to suicide.^(8)^ Depression has recently been described as the main mental disorder in Chinese health workers exposed to COVID-19.^(9)^ healthcare workers depression fluctuates between 12% and 24% and is affected by the health status of countries.^(5,6,10)^

The COVID-19 Care and Isolation Center-Villa Panamericana was a complex designed to accommodate athletes during the Lima 2019 Pan American Games. This center in Peru has been adapted to accommodate and care for approximately 9,000 patients with symptoms or suspected COVID-19. It is the only place in the world that can accommodate both frontline healthcare workers and COVID-19 patients.

These prolonged stays in COVID-19 Care and Isolation Center-Villa Panamericana could affect the well-being of healthcare workers and may promote the development of mental disorders during periods of care since the mental health prevention strategies of the Ministry of Health are not fully prioritizing specific care for healthcare workers that are exposed to COVID-19.^(11)^

## OBJECTIVE

To determine the levels of depression in frontline healthcare workers during the first SARS-CoV-2 outbreak in Peru.

## METHODS

### Study design and setting

A single-center cross-sectional study was designed in the COVID-19 Care and Isolation Center-Villa Panamericana during 2020. It is the Social Security (*EsSalud* - Social Health Insurance Institute, Peru, Americas) health center located in eastern Lima (district of Villa El Salvador), and has an inpatient area for the care and rehabilitation of COVID-19 patients. The COVID-19 Care and Isolation Center-Villa Panamericana also houses about 150 healthcare workers (physicians, nurses, medical technologists, health technicians, and administrative personnel) in a continuous biweekly period with SARS-CoV-2 infection controls at entry and exit. The COVID-19 Care and Isolation Center-Villa Panamericana has two towers, each of which has 20 floors. Each tower has an area for reference, an inpatient area, intensive surveillance unit, X-rays, ultrasound, and laboratory.

### Participants and stay in diagnostic COVID-19 Care and Isolation Center-Villa Panamericana

Frontline healthcare workers who voluntarily agreed to participate in the study were included. Health professionals have a cycle of stay in COVID-19 Care and Isolation Center-Villa Panamericana, they are accommodated for 15 days and checked for COVID-19 at the entrance and exit of the work period with serological tests by immunochromatography IgG/IgM (SafeCare Biotech, Hangzhou, China) followed by conventional reverse-transcriptase polymerase chain reaction (RT-PCR) in case of positive screening. Daily work includes continuous shifts of up to 12 and 24 hours, depending on the type of care provided.

### Survey and data collection

To determine the levels of depression, two specialized professionals surveyed health workers during their rest hours. The survey was conducted in approximately 15 minutes, and interviews were conducted directly in the rest places of the health professionals.

The interviews were conducted between the 2^nd^ and 4^th^ day of the healthcare workers rest. The Montgomery-Asberg Depression Rating scale (MADR-S) translated into Spanish^(12)^ was used and participants signed an informed consent before the survey. The MADR-S is a ten-item diagnostic questionnaire validated in several countries which uses Likert-scale answers and cutoff points are 0 to 6 (depression symptom absent), 7 to 19 (mild depression), 20 to 34 (moderate depression), and >34 (severe depression).

### Data analysis

The data was directly encoded into a data matrix in IBM SPSS v22.0 (Armonk, US), and two authors independently reviewed this database. The initial analysis was performed with descriptive statistics and following the MADR-S guidelines. The Kolmogorov-Smirnov test was used to verify the normality of the data, one-way ANOVA (with Bonferroni post-hoc test) to evaluate the differences between groups and the Spearman correlation test considering a 95% confidence interval and a p value <0.05 as significative. This study was approved by the Ethics Committee of Universidad Norbert Wiener (FCE-RRR-COVID-2020.03-01) under protocol # 0102-2020.

## RESULTS

Of the 96 participants (30±5.6 years), 15 (15.6%) were physicians, 39 (40.6%) were nurses, 14 (14.6%) were medical technologists, and 28 (29.2%) were nurse technicians. There was no significant difference between the age of 54 men (30.7±4.7 years) and 42 women (29.1±4.5 years) (p=0.117). Most of the participants were between 26 and 35 years old (78 [81.3%]), with three to five years of service (61 [63.5%]), and came from Lima (83 [86.5%]). Almost all the participants (89 [92.7%]) lived in urban areas (Table 1).

**Table 1:** Baseline characteristics of COVID-19 Care and Isolation Center-Villa Panamericana healthcare workers with depression levels during the COVID-19 pandemic in Peru

Characteristics	Depression (MADR-S)				p value

	No depression	Mild	Moderate	Severe	
Total	8 (8.3)	35 (36.5)	44 (45.8)	9 (9.4)	0.037
Age group (years)					
≤25	2 (25)	1 (12.5)	4 (50)	1 (12.5)	0.077
26-35	2 (2.6)	31 (39.7)	37 (47.4)	8 (10.3)	
≥36	4 (40)	3 (30)	3 (30)	0 (0)	
Sex					
Woman	5 (5)	15 (35.7)	18 (42.9)	4 (9.5)	0.083
Man	3 (5.6)	20 (37)	26 (48.1)	5 (9.3)	
Profession					
Physician	0 (0)	5 (33.3)	8 (53.3)	2 (13.3)	0.005
Nurse	3 (7.7)	15 (38.5)	18 (46.2)	3 (7.7)	
Medical technologist	1 (7.1)	7 (50)	5 (35.7)	1 (7.1)	
Nurse technician	4 (12.5)	8 (37.5)	13 (40.6)	3 (9.4)	
Length of service					
≤2	3 (13)	9 (39.1)	10 (43.5)	1 (4.3)	0.001
3 to 5	5 (8.2)	23 (37.7)	27 (44.3)	6 (9.8)	
≥6	0 (0)	3 (25)	7 (58.3)	2 (16.7)	
Place of origin					
Lima	8 (9.6)	31 (37.3)	36 (43.4)	8 (9.6)	0.022
Province	0 (0)	4 (30.8)	8 (61.5)	1 (7.7)	

MADR-S: Montgomery-Asberg Depression Rating scale.

**Figure 1:**
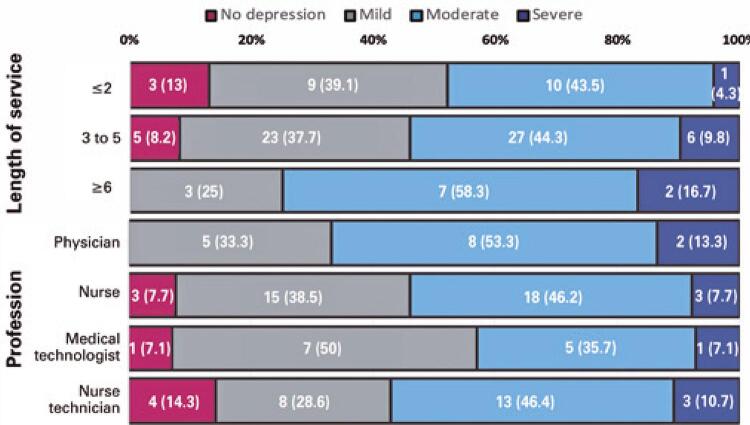
Distribution of depression levels according to the time of service and profession of the Center-Villa Panamericana healthcare workers during the COVID-19 pandemic in Peru. The high rates of depression in physicians and nurses as well as in those who have been working for more than 6 years can be highlighted

A considerable number of participants had symptoms of depression (88 [91.7%]) (Figure 1). Mild, moderate, and severe depression were present in 35 (36.5%), 44 (45.8%), and 9 (9.4%) of the cases, respectively. Half the medical technologists had more symptoms of mild depression, while physicians and nurses reported more severe levels of depression (moderate depression among physicians (8 [53.3%]) and nurses (18 [46.2%]); severe depression among physicians (2 [13.3%]) and nurses (3 [7.7%]), p=0.005). In addition, the study determined greater symptoms of depression according to years of work (moderate depression: three to five years of work (27 [44.3%]) *versus* ≥6 years of work (7 [58.3%]), severe depression three to five years of work (6 [9.8%]) *versus* ≥6 years of work (2 [16.7%]), p=0.001).

Among all participants, 32 (33.3%) had COVID-19, of which 4 (12.5%) were physicians, 9 (28.1%) were nurses, 7 (21.9%) were medical technologists, and 12 (37.5%) were nurse technicians. The study found an association between length of service (p=0.012) and profession (p=0.008) with SARS-CoV-2 infection. Although sex and length of service (p<0.05) showed an association with levels of depression, it was not associated with profession or age group. The median age of the infected participants was 31.7±5.8 (95%CI 29.7 to 33.8), most of them were male (18 [56.3%]) and had worked for three to five years (23 [71.9%]).

Only 4 (12.5%) participants with COVID-19 did not present symptoms of depression. Differences were found between healthcare workers with previous COVID-19 and without disease (p=0.002). Twenty-four (75%) participants showed symptoms of COVID-19 and developed moderate (12 [37.5%]) and severe (3 [9.4%]) symptoms of depression (p=0.041). Six patients required symptomatic treatment for COVID-19, of these 3 (9.4%) participants developed severe depression (Table 2).

**Table 2:** Depression levels in healthcare workers with COVID-19 infection during the pandemic in Peru

Characteristics	Depression (MADR-S)				p value

	No depression	Mild	Moderate	Severe	
COVID-19	4 (12.5)	8 (25)	16 (50)	4 (12.5)	0.033
Symptoms					
Asymptomatic	1 (3.1)	2 (6.2)	4 (12.5)	1 (3.1)	0.041
Symptomatic*	3 (9.4)	6 (18.7)	12 (37.5)	3 (9.4)	
Treatment					
None	2 (6.2)	8 (25)	15 (46.8)	1 (3.1)	0.104
Symptomatic^#^	2 (6.2)	0 (0)	1 (3.1)	3 (9.4)	

* Includes mild and moderate symptoms; ^#^ Symptomatic treatment included management of fever, headache, cough, and nausea. MADR-S: Montgomery-Asberg Depression Rating scale; COVID-19: coronavirus disease 2019.

## DISCUSSION

The present study found that more than 90% of healthcare workers had symptoms of depression, which was worse in physicians and nurses, who manifested levels of moderate and severe depression in ~50% and ~10%, respectively.

Globally, the increased workload due to COVID-19 and limited resources (personal protective equipment, intensive care beds, mechanical ventilation equipment, etc.) leads to increased risk of contagion (personal and family), and the ethical challenge in the decisions of prioritization and fulfillment of activities are generating psychological pressure in the healthcare workers, which frequently lead to the development of a set of mental disorders. Particularly in Peru, there are also political, economic, and social crises during the pandemic, which may further affect the severity of these diseases.

The prevalence of depression among healthcare workers ranges from 1% to 61% depending on the number of participants, and the impact of the pandemic on the affected population.^(8-10)^ Throughout the pandemic, various studies have shown an average prevalence of depression of ~22%. In fact, a recent umbrella review has shown a 24.8% prevalence of depression among healthcare workers during the COVID-19 pandemic worldwide.^(13)^ The findings of this study dramatically disagree with these previous studies as symptoms of depression were present in ~90% (88/96) of the COVID-19 Care and Isolation Center-Villa Panamericana healthcare workers, with the level of mild depression being the most frequent in 36.5% of participants. This marked difference found in Peruvian healthcare workers could be explained by a set of intrinsic (biweekly accommodation in COVID-19 Care and Isolation Center-Villa Panamericana, direct contact with patients with COVID-19, fatigue and stress, lack and reduction of healthcare workers, increase in deaths, insecurity, fear of contagion and transmission, grief due to loss of relatives etc.) and extrinsic factors (lack of personal protective equipment, medications, oxygen and mechanical ventilators, inadequate tests, delayed payment of healthcare workers monthly salaries, underlying social and political problems that keep cases increasing etc.). In addition, the work experience of medical staff is becoming increasingly personal, affecting their family and interpersonal relationships.

Consistent with previous studies,^(6,10,13,14)^ among healthcare workers, physicians and nurses (including nurse technicians) have higher levels of major depression. The severity of depression is likely to be an aggravation of previous mental illness because several studies show many healthcare workers have already experienced symptoms of depression before the pandemic.^(15-17)^As well, the precarious conditions during the COVID-19 lockdown have led to a collapse of the health care system and its professionals.

Recently, a regional analysis of depression showed the highest prevalence in the Middle-East (34.6%), but the analysis did not include studies in South America.^(18)^ It is possible that depression, as well as other mental disorders, are being accentuated in South America due to the syndemic that highlights the epidemiological complexity of COVID-19 and a set of social and political factors, ranging from corruption processes in the acquisition of vaccines (Vacunagate) to the lack of preparation and resources to face the second and third wave of the pandemic.

In this context, mental health of healthcare workers is threatened and may worsen, leading to suicide in the severe cases (*i.e.*, ~10% of cases found in this study). The COVID-19 Care and Isolation Center-Villa Panamericana work system and care center is a unique health center in the world, in which healthcare workers and COVID-19 patients are regularly included in the same milieu, allowing immediate and high-quality health care to be provided. However, due to the potential risk of infection, these processes may lead to the development of mental disorders, increase fear, and reduce the quality of life. Further studies are required to understand the possible long-term mental health effects of the COVID-19 Care and Isolation Center-Villa Panamericana healthcare workers during the pandemic.

On the other hand, greater mental disorders have been evidenced in female nurses from two public hospitals in Peru.^(19)^ Although the prevalence of severe depression in women is slightly higher, the levels of moderate and mild depression are comparable to other healthcare workers. The results of this study showed that among nurses, mild or moderate depression was ≥38%, which is inconsistent with previous studies of depression in Lima (25%) and Cusco (30%).^(19,20)^

An important finding of this study is the evaluation of depression in healthcare workers who had COVID-19, the most affected being nurse technicians and nurses with symptoms of the disease. This study revealed that half of the COVID-19 patients had moderate depression and symptomatic patients had higher levels of depression than asymptomatic patients. To the best of our knowledge, this is the first Peruvian work that evaluated depression in healthcare workers with COVID-19; however, large-scale national-wide studies are needed to understand the prevalence and impact of depression.

This study had the following limitations: first, it focused on the analysis of depression, however, it is necessary to estimate the prevalence of other mental disorders such as anxiety, post-traumatic stress, quality of life etc.; second, there could be differences in the use of different instruments for the analysis of the prevalence of depression in healthcare workers, therefore it is necessary to identify the best instruments. Finally, the results cannot be generalized because health professionals were confined for 15 days in the service place where patients with COVID were hospitalized.

## CONCLUSION

This study clearly demonstrated a high prevalence of depression in COVID-19 Care and Isolation Center-Villa Panamericana frontline healthcare workers during the COVID-19 pandemic in Peru. These results suggest that both physicians and nurses are the main affected and have symptoms of severe depression that must also be a priority within mental health programs during the COVID-19 pandemic, providing organized and useful tools.
